# Construction of *Gossypium* *barbadense* Mutant Library Provides Genetic Resources for Cotton Germplasm Improvement

**DOI:** 10.3390/ijms21186505

**Published:** 2020-09-05

**Authors:** Muhammad Ali Abid, Peilin Wang, Tao Zhu, Chengzhen Liang, Zhigang Meng, Waqas Malik, Sandui Guo, Rui Zhang

**Affiliations:** 1 Biotechnology Research Institute, Chinese Academy of Agricultural Sciences, Beijing 100081, China; abid@caas.cn (M.A.A.); wangpeilin19@126.com (P.W.); taozhu@mail.bnu.edu.cn (T.Z.); liangchengzhen@caas.cn (C.L.); mengzhigang@caas.cn (Z.M.); guosandui@caas.cn (S.G.); 2 Genomics Lab, Department of Plant Breeding and Genetics, Bahauddin Zakariya University, Multan 60000, Pakistan; waqasmalik@bzu.edu.pk

**Keywords:** ethylmethanesulfonate, mutation breeding, mutant bank, germplasm resources, plant architecture, virescent phenotype

## Abstract

Allotetraploid cotton (*Gossypium hirsutum* and *Gossypium barbadense*) are cultivated worldwide for its white fiber. For centuries, conventional breeding approaches increase cotton yield at the cost of extensive erosion of natural genetic variability. Sea Island cotton (*G. barbadense*) is known for its superior fiber quality, but show poor adaptability as compared to Upland cotton. Here, in this study, we use ethylmethanesulfonate (EMS) as a mutagenic agent to induce genome-wide point mutations to improve the current germplasm resources of Sea Island cotton and develop diverse breeding lines with improved adaptability and excellent economic traits. We determined the optimal EMS experimental procedure suitable for construction of cotton mutant library. At M_6_ generation, mutant library comprised of lines with distinguished phenotypes of the plant architecture, leaf, flower, boll, and fiber. Genome-wide analysis of SNP distribution and density in yellow leaf mutant reflected the better quality of mutant library. Reduced photosynthetic efficiency and transmission electron microscopy of yellow leaf mutants revealed the effect of induced mutations at physiological and cellular level. Our mutant collection will serve as the valuable resource for basic research on cotton functional genomics, as well as cotton breeding.

## 1. Introduction

Identification of novel alleles in cotton has taken an immense importance in facilitating the genetic improvement and functional genomics research. Conventional breeding approaches have made enormous contributions to the cultivation of cotton varieties; however, increasing biased manipulation of available genetic variability in cotton germplasm resources has resulted in great loss of genetic potential and increased vulnerability to many insect/pests [1]. This selection pressure has reduced the allelic diversity and hampers the efforts to improve the agronomic traits of cotton and limit our understanding about the molecular mechanisms that respond to environmental stresses and pathogens [2]. In this scenario, broadening the genetic base by induced mutation can diversify and create novel changes in the functional genes to develop more germplasm resources [3]. Various mutagenic techniques have been routinely used in cotton gene function studies like T-DNA insertion [4] and gene silencing approaches [5], but these techniques may be impractical, time consuming, and costly in cotton species owing to transgenic barriers. While induced physical and chemical mutagenesis have been demonstrated as an easy and cost-effective way for wide range of genetic mutation. A variety of physical or chemical mutagenic agents have been used to generate genetic variability in cotton, and the most commonly used agents are fast-neutron, gamma-ray bombardments, and ethylmethanesulfonate (EMS). Use of several ionizing radiations and chemical mutagens can be employed to effectively increase the mutation frequency in *Gossypium* species [6]. Among physical and chemical mutagenic sources, EMS has demonstrated as an excellent mutagenic agent that activates mutagenic potential of crop plants [7]. EMS mutagenesis generates point mutations that are evenly distributed over the whole genome, hence generating large genotypic and phenotypic diversity [1]. EMS effectively replaces the G:C pairing at the nitrogenous base level with any other base pairing through 3-ethyladenine pairing errors bringing 2–10 mutations/Mb of diploid DNA [8]. Mutagenesis in combination with modern genetic tools has the potential to rapidly utilize the genetic variability of targeted loci in cotton for both improving fiber characteristics and abiotic/biotic stress tolerance. EMS-induced mutations in Upland cotton has been used to generate new valuable alleles and traits, leading to development of commercial cultivated varieties [6,9,10,11,12]. Recently, EMS-induced mutant libraries have been constructed in Upland cotton [13] representing beneficial genetic variations. Such collection of mutants represented a broad repertoire of mutants, that may serve as a valuable resource for functional genomic research on complex allotetraploid traits. In EMS-induced mutagenesis, suitable doses of EMS are required to achieve the ideal results. Generally, mutagen doses inducing about 50% lethality (LD50) among M_1_ plants are considered as appropriate dose because of their high mutation frequency [14].

Sea Island cotton (*G. barbadense*) is the second most cultivated cotton species, known for its longer staple and superior fiber quality, with a genome size of 2.2 Gb and high content of repetitive elements [15]. After polyploidization, *G. barbadense* L. evolved to produce a superior fiber quality but show lower diversity than *G. hirsutum*, which is most cultivated and geographically diverse specie. Only few studies had been conducted on *G. barbadense*, despite its superiority in fiber quality over *G. hirsutum*, and accounted for less genetic diversity [16]. Because of its genome complexity, only few genes were identified using the map-based cloning approach. Hence, EMS-based mutagenesis provide reliable source for manipulating genetic mechanism underlying cotton plant architecture, leaf, flower, and fiber related traits is essential for engineering climate-resilient Sea Island cotton varieties. The sympodial and monopodial branches are major determinant of plant architecture and are therefore agronomically important [17]. Leaves are the main source of photo-assimilate in crop plants and photosynthesis is a regulated process that is highly sensitive to environmental stress, as it needs to balance the light energy absorbed by the photosystems with the energy consumed by the metabolic sinks. The chlorophyll (*Chl*) contents of leaves are positively correlated with photosynthetic efficiency and greatly affect the yield and quality of cotton [18]. Recently, EMS-induced mutation resulted in threonine/isoleucine substitution in a tetratricopeptide repeat-like superfamily protein which is responsible for the short fiber phenotype in Upland cotton [19].

In this study, our aim is to improve the high yield breeding potential and generate diversity in *G. barbadense*, therefore we use ISR (*G. barbadense*) genotype as a source material for EMS-induced mutagenesis and develop mutant library. Several types of morphological variations was observed in stable EMS-induced mutants (M_6_ generation) including plant height, internodal distance, number of monopodial branches, number of sympodial branches, compact and spreading branches, first fruit branch node, flower size, length of floral style, number of bolls, boll size, boll shape, leaf color, pigmented leaf, leaf shape, petal spot, yellow leaves, and fiber length. Then we choose yellow leaf mutant line and conducted whole genome sequencing, determined chlorophyll contents, and ultrastructure of chloroplast which reflect the quality of mutant library. Our *G. barbadense* mutant library provides an enriched resource for forward and reverse genetic analysis of various gene families and annotating the important genes, related to plant architecture, yield, and fiber quality traits. Additionally, the identification and analysis of plant gene mutants is the basis of gene function research [20] and the establishment of our mutant library can provide resources for molecular biology research.

## 2. Results

### 2.1. Determination of Lethal Dose of EMS Treatment

**Figure 1:**
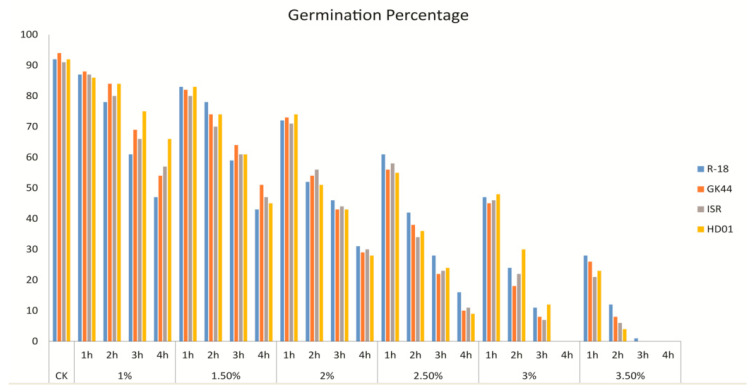
Determination of LD50 of ethylmethanesulfonate (EMS) treatment on different genotypes of cotton.

EMS induced different mutagenic effects on the growth and development of different cotton species and varieties, therefore it is inevitable to determine the optimum dose of EMS before treating large-scale experiment. In this study, lethal dose (LD50) of EMS was determined by germination percentage of ISR, HD01 (*G. barbadense* L.), and R18, GK44 (*G. hirsutum* L.) genotypes using different concentration of EMS i.e., 1.0%, 1.5%, 2.0%, 2.5%, 3.0%, 3.5% (*v*/*v*) and treatment time (1–4 h) using distilled water as a control (Figure 1). The germination rate (%) of control group of R18, GK44, ISR, and HD01 was 92, 94, 91, and 92 respectively. Significant differences in germination rate from 1–4 h of treatment time and 1% to 3.5% of EMS doses were observed in all genotypes. Seed germination rate decreased with the increase in EMS concentration and treatment time. Increased EMS dose and treatment time resulted in increased mortality rate of all tested genotypes indicating higher doses and longer treatment time was enough to kill the seeds. At 2% dose of EMS and 2 h of treatment time, germination rate reduced to nearly 50% for these genotypes which was closest to LD50.

**Figure 2:**
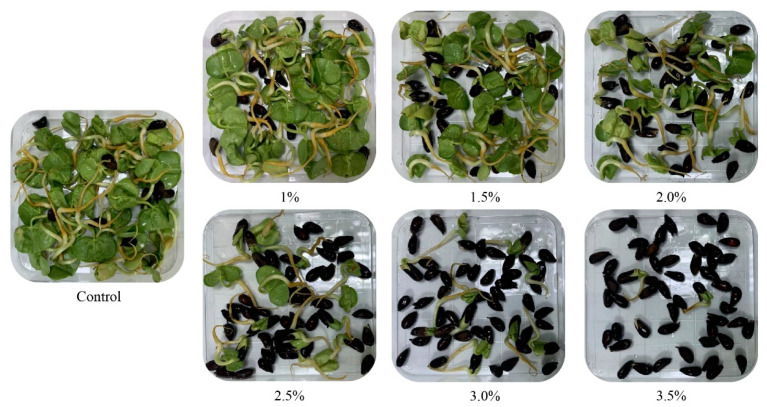
Germination rate of ISR seeds under different concentrations of EMS.

Genotype ISR was selected for large-scale mutagenic treatment and mutant library construction using the dose and treatment time estimated in preliminary tests. Germination rate of ISR in different concentration of EMS treated for 2 h have been presented in Figure 2. Therefore, for field experiment 2% of EMS dose and 2 h of treatment time was selected as the optimized EMS treatment conditions for cotton.

### 2.2. EMS-Induced Abundant Phenotypic Mutations

**Figure 3:**
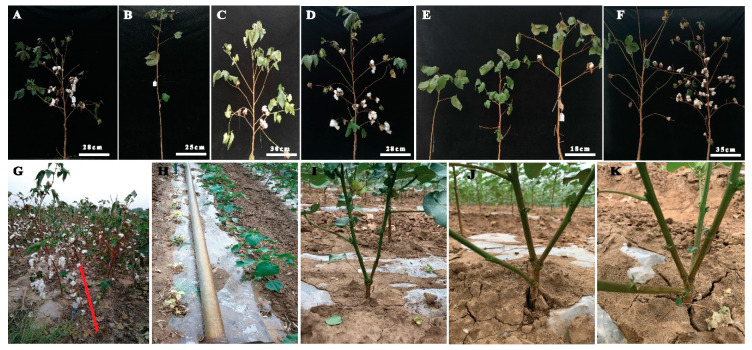
Phenotypic analysis of plant architecture in EMS-induced mutants. (**A**) Wild type plant architecture, (**B**) mutant phenotype without fruiting branch, (**C**) medium height with spreading branches, (**D**) high yielding mutant as compared to wild type, (**E**) comparison of fruit branch pattern including without fruiting branch, compact and short fruit branch, longer fruit branches, (**F**) Longer and spreading branches with different boll bearing capacity, (**G**) comparison of mutant lines high and low seed cotton yield, (**H**) comparison of yellow leaf plants with green leaf plants, (**I**) mutant plant with two main stems, (**J**) mutant plant with three main stems, (**K**) mutant plant with four main stems.

For field experiment, about 3 kg healthy seeds of ISR were treated with EMS and planted in the field. The phenotypic evaluation showed that EMS induced plentiful chimeric plants (361 out of about 5000) with frequency of phenotypic variations reaching to 7.22% in M_1_ generation. EMS-induced mutagenesis leads to the development of stable mutants at M_6_ generation with considerable variations in plant height, internodal distance, number of monopodial branches, number of sympodial branches, compact and spreading branches, first fruit branch node, flower size, length of floral style, number of bolls, boll size, boll shape, leaf color, pigmented leaf, leaf shape, petal spot, yellow leaves, and fiber length. We found EMS treatment had effectively induced genetic mutation in our cotton mutant library and produced mutant phenotypes that were mostly exhibited in the whole plant architecture, leaf, flower, and cotton boll where the traits can be easily observed. A list of most obvious variations in morphological traits of mutant lines is presented in supplementary Table S1. Mutant lines displayed several types of variations in plant architecture e.g., short plant height, without fruiting branch, fruit branch at 3rd node (Figure 3).

**Figure 4:**
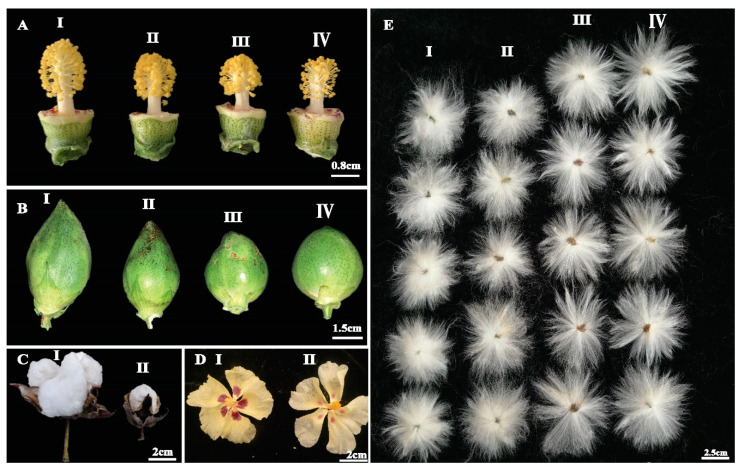
Phenotypic analysis of floral traits in EMS-induced mutants. (**A**) Comparison of wild type (II) and mutant lines (I, III, and IV) with varying length of flower style and anther numbers, **(B**) comparison of wild type (II) with mutant lines (I, III, and IV) with different boll size and shape ranging from oblong shape to nearly round boll, (**C**) evaluation of fully mature cotton boll with big boll size in wild type (I) and small boll size in mutant line (II), (**D**) comparison of wild type (I) and mutant line (II) for size and intensity of petal spot, (**E**) comparison of fiber length with I and II as mutants with short fiber length, III represent as fiber length of wild type, IV represent mutant with longer fiber length.

A mutant line was found with relatively more boll numbers as compared to wild type (Figure 3D). Branching pattern was the most obvious phenotype, ranging from without fruiting branch to longer fruiting branch with varying boll bearing capacity, compact and spreading type fruiting branch pattern, mutant lines with two, three and four main stems in mutant plants. Other architectural traits of mutant lines include significant variations in plant height, seed cotton yield, virescent phenotype, and multi stem phenotype. Variations in the floral trait mostly include size and shape of flowers and cotton bolls (Figure 4). Developing bolls varied from oblong shape to nearly round shape. In addition, petal spot pigmentation varied in size and intensity of color, length of floral style and number of anthers significantly varied among mutant lines. We had also observed contrasting fiber length among mutant lines of our library, variation in fiber length ranged from shorter fiber length to longer fiber length as compared to wild type.

**Figure 5:**
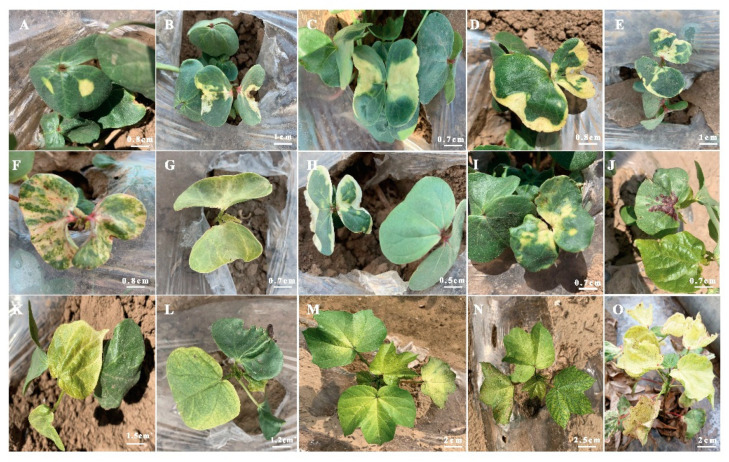
Yellow and yellow-green leaves of EMS-induced mutants (**A**–**J**) cotyledon leaves with different shades and positions of yellow color and anthocyanin pigments, (**K**–**O**) young true leaves with different shades of yellow and yellow-green color.

In the phenotypic analysis of leaf color, we screened several shades of yellow colored cotyledon leaves, anthocyanin pigmented cotyledon leaves and virescent (yellow, yellow-green leaf color) true leaves as compared to green leaves of wild type (Figure 5). Such mutants with virescent leaf pattern were identified in M_2_ generation, which exhibited yellow leaf color at emergence and then gradually turned to green. Now our mutant library contained stable virescent leaf phenotype that represent most distinguished variation in leaf color and plant height. Most of yellow mutants remained stunted and did not develop flowers, while yellow-green mutants showed intermediate height and developed fertile flowers and produced seeds. These phenotypes represent the potential of EMS-induced mutagenesis in cotton for plant architecture and seed cotton yield improvement.

### 2.3. Genome-Wide Analysis of EMS-Induced SNPs in Yellow Leaf Mutant Line

In order to determine the point mutation density, we performed genome-wide analyses of most obvious yellow leaf (virescent) mutant line (YL01) from the EMS mutant library by re-sequencing. Based on the high-quality whole-genome re-sequencing that meets the specifications for further EMS mutant analysis, the genome-wide EMS-induced mutation distribution, mutation sites number, and SNPs annotation were analyzed (Figure S1, Table 1 and Table 2). Genome-wide mutation analysis illustrated that EMS-induced SNPs were generally distributed in the cotton mutant genome with the ideal mutation uniformity. In addition, number and density of EMS SNPs on 26 chromosomes revealed a total of 1,406,370 SNPs, among which 387,577 (27.5%) were newly arisen SNPs in mutant line YL01. Further analysis was carried out based on these newly arisen EMS SNPs with high-mutation density of 181.7 per Mb (Table 1). In addition, genome-wide annotation statistics of EMS SNPs indicated 81.1% of SNP occurred in the intergenic region, while nearly 9.03% were located on downstream/upstream of genes (*cis* elements) and 9.16% in genic region with 1.60% in functional non-synonymous while very few SNPs (0.20%) were found in the gene splicing and UTR regions (Table 2). This data suggested that the phenotypic mutants could be mostly caused by mutation on the functional genic and *cis* element regions.

### 2.4. Yellow Leaf Mutants with Reduced Chlorophyll Contents

**Figure 6:**
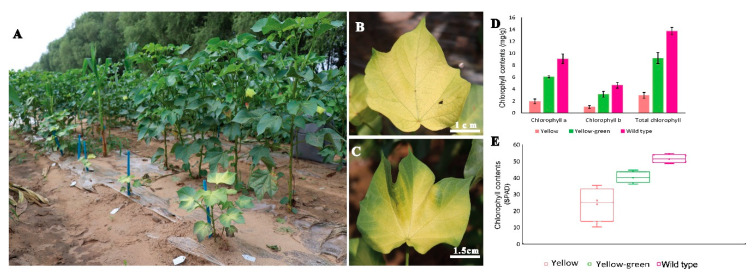
Yellow and yellow-green leaf mutants with reduced chlorophyll contents (**A**) yellow and yellow-green leaf mutants with reduced plant height as compared to wild type plants, (**B**) Pure yellow leaf color, (**C**) yellow-green leaf color, (**D**) chlorophyll contents of wild type, yellow and yellow-green leaf mutants, (**E**) chlorophyll contents of wild type, yellow and yellow-green leaf mutants using SPAD meter.

Virescent mutants displayed yellow and yellow to green leaf color which gradually turned to green upon maturity (Figure 6). The yellow to green virescent mutants gradually grow and develop flowers and bolls. Significant difference was observed in chlorophyll a, chlorophyll b, and total chlorophyll contents from 4th leaf stage of yellow mutants, yellow-green mutants, and normal green leaves of wild type plants. Chlorophyll contents were reduced in yellow and yellow to green mutants as compared to wild type. The yellow leaf mutants had the least chlorophyll contents (Figure 6D). To further validate the chlorophyll contents of yellow, yellow-green mutants, and wild type plants, we used SPAD meter to determine chlorophyll contents values from yellow, yellow to green and wild type plants in three repeats. SPAD values significantly differentiates the yellow and yellow-green mutants from the wild type plants (Figure 6E). Yellow leaf mutants had average SPAD value of 14.2, yellow-green mutants had average SPAD value of 31.9, while wild type plants had average SPAD value of 48.7. This data suggested that yellow and yellow-green virescent mutants had impaired photosynthetic system with significantly reduced chlorophyll contents as compared to wild type.

### 2.5. EMS Treatment Resulted in Disrupted Chloroplast Structure

**Figure 7:**
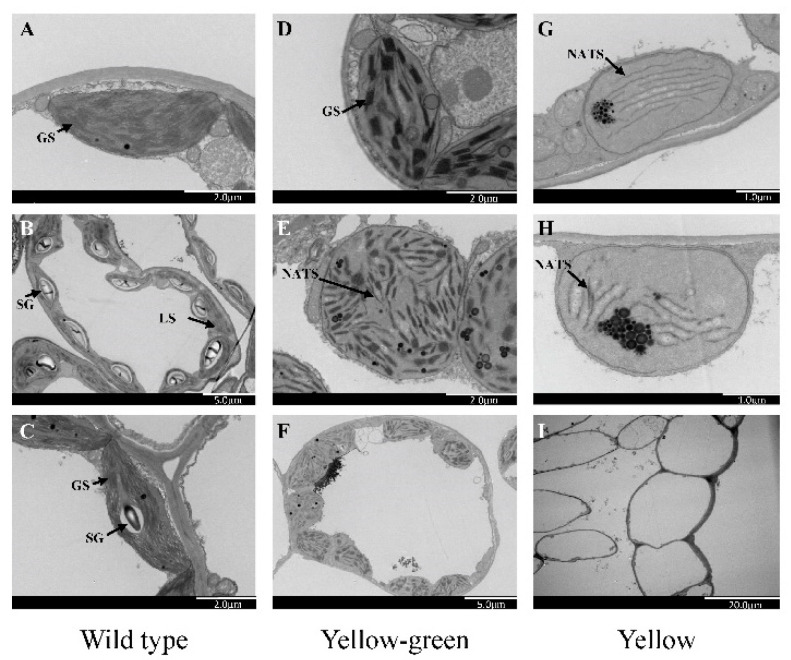
Transmission electron microscopy of the chloroplast ultrastructure, (**A**–**C**) Electron micrographs showing mesophyll cells and ultrastructure of chloroplast in wild type, (**D**–**F**) yellow-green leaf mutants, (**G**–**I**) yellow leaf mutants. GS, granal stack; SG, starch grain; NATS, non-appressed thylakoid stacking; LS, lamellar structure.

In order to determine whether the yellow leaf and yellow-green leaf mutation affected chloroplast development, the ultrastructure of chloroplasts of both mutants and wild-type plants at 3–4 leaf stage was observed by TEM. The wild type had several large functional granal stacks (Figure 7A–C), whereas abnormal and non-appressed thylakoid stacking was observed in both types of mutants, with only a few rare, granal stacks observed in yellow-green mutants (Figure 7D–F). TEM also demonstrated a significantly lower chloroplast number per cell in the yellow leaf mutant’s mesophyll cells than those in wild type. In addition, in most of mesophyll cells chloroplast number in yellow-green mutants were also less than wild type. Most of the chloroplasts in the yellow mutant exhibited fewer lamellar structures than the wild-type, no granum lamella, or stroma lamella.

## 3. Discussion

Chemical mutagenesis has demonstrated to be an effective tool to create a wide range of phenotypic variations in both diploid and tetraploid *Gossypium* populations [7,9,11]. EMS is frequently used as a chemical mutagen for both forward and reverse genetic studies in crop plants. Usually EMS mutagens induced point mutations (single nucleotide changes) while physical mutagens (gamma radiations) mostly cause deletions (chromosomal aberrations) that result in lethal or negative effects [7,21]. However, there are also successful examples of utilizing gamma rays-induced mutations in cotton improvement programs [7,22,23]. In this study, we demonstrated the potential of EMS-induced mutagenesis to create a vast mutant library of *G. barbadense* and induced genetic variability can be used to identify gene-regulating important phenotypes. Such strategies can also be efficiently used in cotton improvement programs to develop elite lines by manipulating architectural and fiber quality traits. The 2% *v*/*v* EMS treatment for 2 h had reduced seed (M_0_) viability to nearly 50% but did not appear to impact viability of further generations. Mutant library included several stable mutant lines of M_6_ with range of diverse phenotypes including plant height, internodal distance, number of monopodial branches, number of sympodial branches, compact and spreading branches, first fruit branch node, flower size, length of floral style, number of bolls, boll size, boll shape, leaf color, pigmented leaf, leaf shape, petal spot, yellow leaves, and fiber length (Figure 3, Figure 4 and Figure 5). This mutagenized population might serve as an important source of genetic and phenotypic studies. For example, mutant lines with increased number of bolls and improved fiber length as compared to wild type plant, might be used as germplasm resources for cotton improvement programs. Genome-wide analyses of EMS-induced SNPs in virescent mutant line YL01 by resequencing revealed the ideal quality of the cotton EMS mutant library with the high and appropriate mutation density (Table 1). Recently published EMS mutant library of Upland cotton [13] showed 85% of EMS SNPs occurred in intergenic region compared to 81.8% of our Sea island mutant library. In addition, our library quality was further assessed by taking into account 9.16% of the effective functional mutation in genic region compared to 8.3% of Upland cotton mutant library. In upstream/downstream region of genes, 9.03% of EMS SNPs were induced in our library compared to nearly 6% in Upland cotton mutant library (Table 2). Such differences in the ratio of EMS SNPs between Sea island and Upland cotton libraries might be due to different genome size and different treatment doses of EMS and treatment time.

As photosynthetic efficiency of green leaves in cotton serves as important indicator for high yield, therefore we used yellow leaf phenotype and determined abnormal chloroplast structure among such mutants. Several virescent mutants affecting chloroplast development have been recognized in cotton. Virescent mutants are yellow colored leaves gradually turned to green and described to be controlled by recessive genes [24,25]. Our study demonstrated that majority of the virescent mutants, the yellow and yellow-green mutants were predominantly expressed in the initial stage of growth, mature leaves of the mutants gradually became green and barely distinguishable from leaves of normal plants. The yellow and yellow-green color affected plant development drastically (Figure 6) leading to reduced plant height, number of bolls per plant, boll weight, and fiber quality. As chlorophyll content of the yellow and yellow-green leaf mutant lines was significantly lower, therefore vigor of the virescent mutant plants was lower than wild type. TEM indicated chloroplast ultrastructure of yellow and yellow-green mutants, thylakoid structures were changed and therefore development of chloroplasts was limited and delayed (Figure 7). Number of grana and stroma thylakoids in chloroplast were less and arranged in a chaotic way in younger leaves. Virescent mutations thus appeared to affect thylakoid stacking resulting in the reduction in chlorophyll content. Molecular characterization of genes responsible for such functional traits identified in our mutant populations will yield new insight into our understanding of functional genomics in cotton.

## 4. Materials and Methods

### 4.1. Plant Material and Preliminary Mutagenic Treatment

Seeds of commercially grown cotton genotype ISR, HD01 (*Gossypium barbadense* L.) and R18, GK44 (*Gossypium hirsutum* L.) were obtained from Biotechnology Research Institute, Chinese Academy of Agricultural Sciences, Beijing. Cotton seeds were delinted with concentrated H_2_SO_4_ and presoaked in distilled water for 16 h prior to EMS treatment. To determine the lethal dose (LD50) of EMS, we conducted germination percentage test of these genotypes. Seeds of each genotypes were soaked in six treatments of EMS doses at 1.0%, 1.5%, 2.0%, 2.5%, 3.0%, 3.5% (*v*/*v*) for 1–4 h with distilled water used as control. Soaked seeds of each genotype in each treatment were incubated at room temperature with mild shaking (50RPM) for respective period of time. After treating with EMS, all treatments were rinsed with tap water several times to remove residual reagent, air dried and sowed on culture plates with Whatman filter paper. Seeds were germinated in growth chamber with controlled light 16 h light/8 h dark period at 28 °C. After 10 days of sowing, germination percentage were counted for each treatment and each genotype. To estimate the mutagenic dosage for subsequent experiments and to validate the results, the optimum dose of EMS needs to be pretested in the experiment. The germination percentage is calculated after ten days using the number of seeds that normally germinate divided by the total number of seeds sown, multiplied by 100%. The germination of seeds is designated by the radicle length reaching 1/2 of the seed length. Finally, the treatment that resulted in median LD50 was selected as the optimum dose for subsequent experiments.

### 4.2. EMS Treatment and Phenotypic Evaluation of Mutagenic Population

Three kilograms of healthy seeds (M_0_) of ISR were treated with aqueous solution of EMS at 2.0% (*v*/*v*) for two hours followed by rinsing with tap water several times. The mutagenic treatment process was similar to that of the pre-experiment, including delinting the seeds, presoaking, treating the seeds in EMS solution. Treated seeds were planted in experimental base camp of Biotechnology Research Institute in Pinggu, Beijing. Untreated seeds were planted as control. Nearly 5000 mutagenic (M_1_) plants were screened for viable plants and each plant was observed for phenotypic variation including all cotton developmental stages. The selfed seeds from each viable M_1_ plant (total of 361) were used to grow M_2_ lines with 30–35 plants in each line. From each M_2_ line flowers of fertile plants were selfed to develop non-contaminated seeds and similarly get subsequent generations M_3_–M_6_. These mutant generations were observed for variation in comparison with control. Phenotypic evaluation were based on plant height, internodal distance, number of monopodial branches, number of sympodial branches, compact and spreading branches, first fruit branch node, flower size, length of floral style, number of bolls, boll size, boll shape, leaf color, pigmented leaf, leaf shape, petal spot, yellow and yellow-green leaf color (virescent mutants), and fiber length.

### 4.3. Whole-Genome Resequencing of Bulked DNA

For each pool, genomic DNA were extracted from the fresh leaf tissue of yellow leaf mutant line YL01 (Yellow Leaf 01) and the ISR wild type plants using DNeasy Plant Mini Kit (Qiagen-Dusseldorf, Hilden, Germany). The equal weight of fresh leaves (~0.05 g) were used for genomic DNA extraction and used for further pool sequencing. After that, the genomic DNA was fragmented by ultrasonic treatment. A sequencing library was constructed with an insert size of 400–500 bp for a single index according to the protocol of KAPA Hyper Prep Kit (Illumina^®^ platforms, San Diego, CA, USA). The indexed DNA samples of each pool were then purified using a silica membrane column, followed by size-selection agarose gel electrophoresis (Bluepippin, Beverly, MA, USA). The DNA library of each pool was loaded into one lane using the Illumina Hiseq 4000 system. In total, 100 individuals (50 yellow leaves, and 50 green leaves of YL01 and ISR wild type, respectively) were sequenced, generating 150-bp paired-end reads. Alignment against the reference genome sequence (HAU releases build v2_a1 pseudomolecules of sea-island cotton) was performed using BWA software v0.7.17 [26], followed by SNPcalling using Bcftools v0.1.19 [27]. The resulted SNPs were filtered by a minimum depth threshold of 50 reads in each sample. SNPs with wild genotype were regarded as newly arisen mutations in the yellow leaf mutants. Possible SNP affections (e.g., synonymous or missense variants) were annotated using SnpEff v4.3i [28].

### 4.4. Determination of Photosynthetic Pigments

Fresh leaves of cotton yellow, yellow-green leaf mutants and wild type plants were collected at 6–7 true leaves stage and used to determine chlorophyll contents (*chl a*, *chl b*, and *total chl*). Briefly, for chlorophyl extraction, 0.5 g of leaf sample was ground in 80% of acetone solution followed by filtration through Whatman #1 paper. The absorbance of filtrate was recorded at 663 nm and 644 nm. The chlorophyll *a*, chlorophyll *b*, and total chlorophyll contents were calculated according to the method described by [29] with some modifications. For accurate determination of chlorophyll contents, third and fourth leaves (representing the plant canopy) of field-grown plants were considered to be the most suitable for chlorophyll measurement in yellow, yellow-green, and green leaves plants. The chlorophyll contents of these leaves was also used for SPAD chlorophyll meter reading (SCMR) by SPAD-502 Plus meter (Konica Minolta Co. Ltd., Chiyoda-ku, Japan) as described by [30]. Measurements were recorded in six independent replications each with three technical replications.

### 4.5. Transmission Electron Microscopy

Leaf samples of yellow leaf mutants and wild type were prepared for transmission electron microscopy (TEM) from the leaf blade of 4th leaf. Transverse leaf sections were vacuumed and fixed in 2.5% glutaraldehyde in a phosphate buffer at 4 °C for 4 h. The samples were then dehydrated in a series of graded ethanol and critical point drying was done using liquid CO_2_ in a critical-point drier Bal Tec CPD 030 and 15 nm gold coated on aluminum stubs in a Sputte Coater Bal-Tec SCD 005. The samples were visualized with a Hitachi S-3500N scanning electron microscope. In order to count the number of chloroplasts and to survey the structure of the chloroplasts, 20 cells were examined from each sample.

## 5. Conclusions

Cotton is amenable to chemicals and radiation-based mutagenesis and had been used to successfully increase genetic variability. However, in the past, mutation breeding had played a less significant role in the improvement of cotton. Here we demonstrated the construction of mutant library with considerable amount of genetic diversity which could be used as germplasm resource for cotton (*G. barbadense*) breeding program. Current study determines and verifies the optimal EMS-based procedure that is suitable for cotton (*G. barbadense*) mutant library construction, the collection is consisted of a broad repertoire of mutants, which is a valuable resource for cotton genetic improvement as well as basic genetic research and functional genomics underlying complex traits.

## Figures and Tables

**Table 1:** Genome-wide EMS-induced SNP numbers and mutation density in YL01.

Chromosome	No. of SNPs	Chromosome Length	Mutation Density (per Mb)
A01	57,780	115,637,255	499.7
A02	36,446	100,057,689	364.2
A03	5770	105,315,579	54.8
A04	6089	81,554,553	74.7
A05	10,435	102,776,486	101.5
A06	63,929	115,140,250	555.2
A07	14,189	92,880,876	152.8
A08	11,397	119,882,356	95.1
A09	7256	77,927,517	93.1
A10	6575	110,302,803	59.6
A11	44,148	113,101,708	390.3
A12	6030	102,106,374	59.1
A13	7703	109,233,746	70.5
D01	8891	62,811,768	141.5
D02	6002	67,659,327	88.7
D03	4275	50,986,338	83.8
D04	18,630	52,479,919	355.0
D05	12,870	63,323,194	203.2
D06	2771	62,964,862	44.0
D07	4960	56,457,815	87.9
D08	10,355	65,987,480	156.9
D09	11,188	51,526,817	217.1
D10	5313	65,706,892	80.9
D11	14,493	68,311,261	212.2
D12	3044	58,873,029	51.7
D13	7038	60,343,961	116.6
Overall	387,577	2,133,349,855	181.7

**Table 2:** EMS-induced SNP annotation summary of yellow leaf mutant (YL01).

Genomic Region	No. of SNP	Ratio (%)
Intergenic	317,078	81.81
Upstream/downstream	34,991	9.03
Upstream	21,048	5.43
Downstream	13,943	3.60
Genic	35,508	9.16
Intronic	20,080	5.18
Exonic	15,428	3.98
Synonymous	4380	1.13
Non-synonymous	6194	1.60
Stop gain	133	0.03
Stop loss	11	0.00
UTR 5′	1590	0.41
UTR 3′	2354	0.61
Splicing	766	0.20

## Supplementary Materials

Supplementary Materials can be found at https://www.mdpi.com/1422-0067/21/18/6505/s1.

## Abbreviations

EMS	Ethylmethanesulfonate
LD50	Lethal Dose at 50%
TEM	Transmission electron microscopy
SNP	Single nucleotide polymorphism
