# Supplementary material for: Construction of Gossypium barbadense Mutant Library Provides Genetic Resources for Cotton Germplasm Improvement
Source: Int J Mol Sci. 2020 Sep 5;21(18):6505. doi: 10.3390/ijms21186505 (PMC7554686; doi:10.3390/ijms21186505)
Supplement: Supplementary file 1 [file ijms-21-06505-s001.zip › Figure S1.pdf]

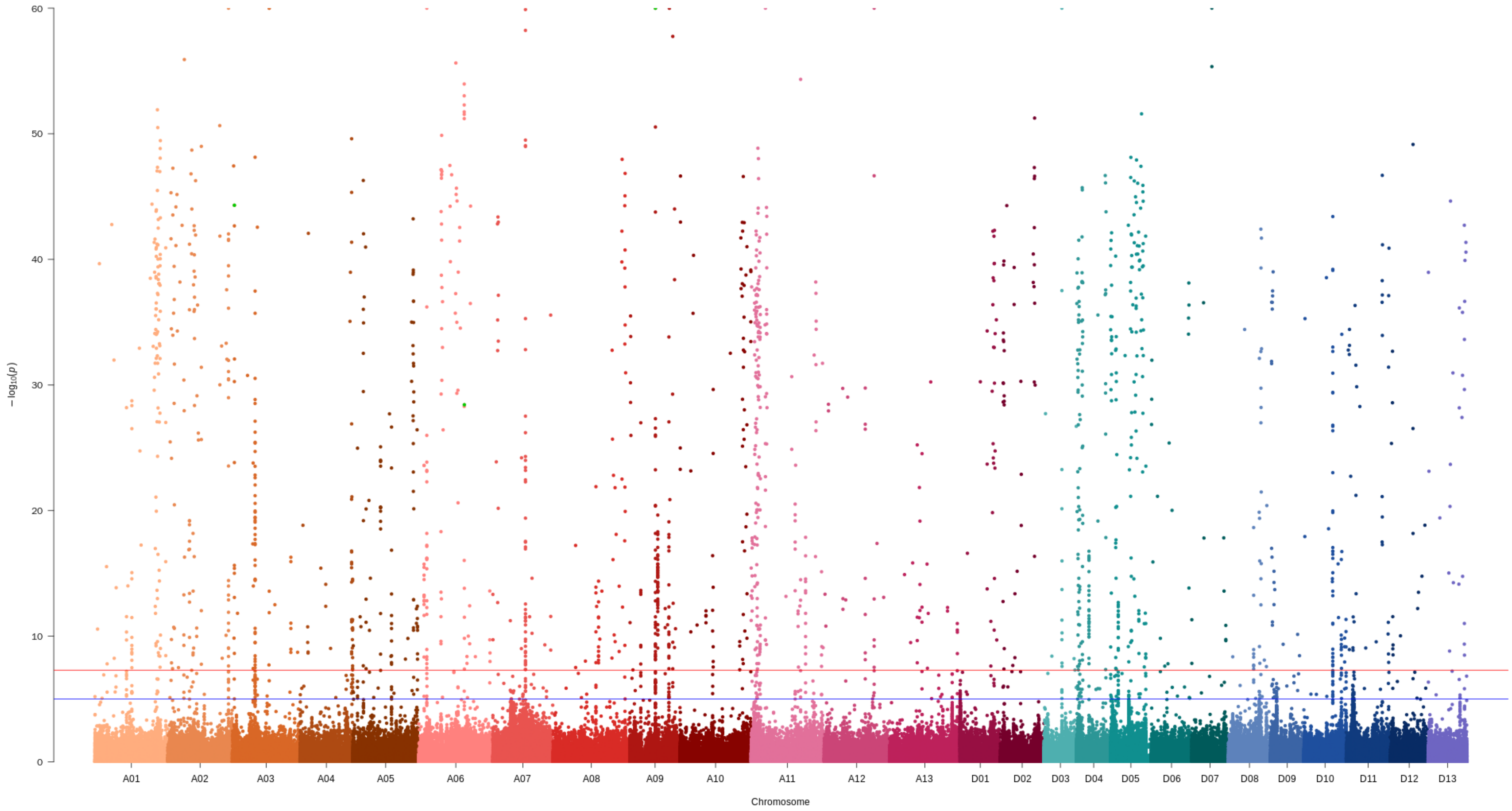

Figure 1. Whole Genome EMS induced SNPs distribution. Manhattan plot was developed using SNP position on each chromosome. After filtering process, we used sequencing data for statistical tests. Using Fisher's Exact Test to calculate *p* value of each variant, we plotted *p* value by using –log10 (*p* value) and chromosomal position as y-value and x-value, respectively. Significant SNPs overlapping with annotated gene regions were selected.
